# Characterization of Conserved Toxicogenomic Responses in Chemically Exposed Hepatocytes across Species and Platforms

**DOI:** 10.1289/ehp.1409157

**Published:** 2015-07-14

**Authors:** Nehme El-Hachem, Patrick Grossmann, Alexis Blanchet-Cohen, Alain R. Bateman, Nicolas Bouchard, Jacques Archambault, Hugo J.W.L. Aerts, Benjamin Haibe-Kains

**Affiliations:** 1 Integrative systems biology, Institut de Recherches Cliniques de Montréal, Montreal, Quebec, Canada; 2 Department of Medicine, University of Montreal, Montréal, Quebec, Canada; 3 Department of Biostatistics & Computational Biology, Dana-Farber Cancer Institute, Boston, Massachusetts, USA; 4 Department of Radiation Oncology, Dana-Farber Cancer Institute, Brigham and Women’s Hospital, Harvard Medical School, Boston, Massachusetts, USA; 5 Bioinformatics, Institut de Recherches Cliniques de Montréal, Montreal, Canada; 6 Department of Human Genetics, McGill University, Montreal, Quebec, Canada; 7 Molecular Biology of Neural Development, Institut de Recherches Cliniques de Montréal, Montreal, Canada; 8 Laboratory of Molecular Virology, Institut de Recherches Cliniques de Montréal, Montreal, Quebec, Canada; 9 Department of Radiology, Dana-Farber Cancer Institute, Brigham and Women’s Hospital, Harvard Medical School, Boston, Massachusetts, USA; 10 Princess Margaret Cancer Centre, University Health Network, Toronto, Ontario, Canada; 11 Medical Biophysics Department, University of Toronto, Toronto, Ontario, Canada

## Abstract

**Background:**

Genome-wide expression profiling is increasingly being used to identify transcriptional changes induced by drugs and environmental stressors. In this context, the Toxicogenomics Project–Genomics Assisted Toxicity Evaluation system (TG-GATEs) project generated transcriptional profiles from rat liver samples and human/rat cultured primary hepatocytes exposed to more than 100 different chemicals.

**Objectives:**

To assess the capacity of the cell culture models to recapitulate pathways induced by chemicals *in vivo*, we leveraged the TG-GATEs data set to compare the early transcriptional responses observed in the liver of rats treated with a large set of chemicals with those of cultured rat and human primary hepatocytes challenged with the same compounds *in vitro*.

**Methods:**

We developed a new pathway-based computational pipeline that efficiently combines gene set enrichment analysis (GSEA) using pathways from the Reactome database with biclustering to identify common modules of pathways that are modulated by several chemicals *in vivo* and *in vitro* across species.

**Results:**

We found that some chemicals induced conserved patterns of early transcriptional responses in *in vitro* and *in vivo* settings, and across human and rat genomes. These responses involved pathways of cell survival, inflammation, xenobiotic metabolism, oxidative stress, and apoptosis. Moreover, our results support the transforming growth factor beta receptor (TGF-βR) signaling pathway as a candidate biomarker associated with exposure to environmental toxicants in primary human hepatocytes.

**Conclusions:**

Our integrative analysis of toxicogenomics data provides a comprehensive overview of biochemical perturbations affected by a large panel of chemicals. Furthermore, we show that the early toxicological response occurring in animals is recapitulated in human and rat primary hepatocyte cultures at the molecular level, indicating that these models reproduce key pathways in response to chemical stress. These findings expand our understanding and interpretation of toxicogenomics data from human hepatocytes exposed to environmental toxicants.

**Citation:**

El-Hachem N, Grossmann P, Blanchet-Cohen A, Bateman AR, Bouchard N, Archambault J, Aerts HJ, Haibe-Kains B. 2016. Characterization of conserved toxicogenomic responses in chemically exposed hepatocytes across species and platforms. Environ Health Perspect 124:313–320; http://dx.doi.org/10.1289/ehp.1409157

## Introduction

Humans are exposed to a variety of toxic chemicals and have access to a wide array of drugs, each of which has the potential to cause short- and long-term adverse effects, including lethality. From an environmental health perspective, it is important to find a strong connection between toxic substances and human disease susceptibility, thereby elucidating molecular mechanisms of toxicity.

Although animal models are currently the gold standard in evaluating risk and predicting adverse human health effects, they require considerable time and resources, and the use of animal models also raises ethical issues (Bissell et al. 2001; Greaves et al. 2004; Hebels et al. 2014; Kola and Landis 2004; Metushi and Uetrecht 2014; Suter et al. 2011). For these reasons, several efforts have been made to minimize the use of animals in toxicology (http://www.alttox.org) and to develop robust *in vitro* models predictive of toxicity in humans (Abbott 2005). A European initiative, the Registration, Evaluation, Authorization and Restriction of Chemicals (REACH) legislation, suggests the use of high-throughput “omics” technologies, such as genome-wide gene expression profiling, to find alternatives to animal testing. The REACH legislation states:

The Commission, Member States, industry and other stakeholders should continue to contribute to the promotion of alternative test methods on an international and national level including computer supported methodologies, *in vitro* methodologies, as appropriate, those based on toxicogenomics, and other relevant methodologies.” (http://www.reachonline.eu/REACH/EN/REACH_EN/preamble1.html)

Multiple studies have used gene expression profiles to characterize toxicogenomic responses (Afshari et al. 2011; Chen et al. 2012; Ellinger-Ziegelbauer et al. 2008; Nuwaysir et al. 1999). To confront chemical-induced cellular stress, the biological system executes a transcriptional control over several signaling pathways (Grinberg et al. 2014; Kier et al. 2004). Because the liver plays a primordial role in detoxification and is a major site of frequent chemical-induced injuries, it has been extensively studied in toxicogenomics. Recently, the Japanese government and the pharmaceutical industry joined forces to create and make publicly available the largest toxicogenomic database to date: the Toxicogenomics Project–Genomics Assisted Toxicity Evaluation system (TG-GATEs) (Uehara et al. 2010, 2011). The TG-GATEs consortium tested ~ 150 chemicals in different models, including primary human and rat hepatocytes as well as rat liver and kidney *in vivo* models (Uehara et al. 2010, 2011). The experimental design and gene expression profiles were made publicly available through the EBI ArrayExpress database (http://www.ebi.ac.uk/arrayexpress/) (Brazma et al. 2003). Different studies used this large toxicogenomic data set to identify predictive biomarkers of hepatocarcinogenicity (Caiment et al. 2014; Yamada et al. 2013), phospholipidosis (Hirode et al. 2008b), and coagulopathy (Hirode et al. 2008a). However, despite the availability of these valuable data, one of the main challenges of toxicogenomics is that it remains unclear whether animal studies can be efficiently replaced by *in vitro* testing to identify key biological pathways induced by hepatotoxic chemicals.

In this study, we performed a large-scale comparative analysis of the TG-GATEs data from rat liver samples (referred to as RLV) and from cultured rat and human primary hepatocytes (referred to as PRH and PHH, respectively) in order to *a*) identify conserved transcriptional responses induced by chemicals across species and between *in vitro* and *in vivo* systems, and *b*) characterize the early response pathways linked to toxicity in both rat *in vivo* and rat/human *in vitro* experiments. Building upon the recent study by Iskar et al. (2013), which showed that certain drugs affected modules of coexpressed genes conserved across a small set of three human cancer cell lines and rat liver samples, we developed a new pathway-based approach that combines gene set enrichment analysis (GSEA) and biclustering to efficiently integrate large-scale toxicogenomic data across different species. Our analysis showed that chemicals affect a set of conserved pathways linked to chemical-induced toxicity across species and experimental platforms.

## Materials and Methods

**Figure 1:**
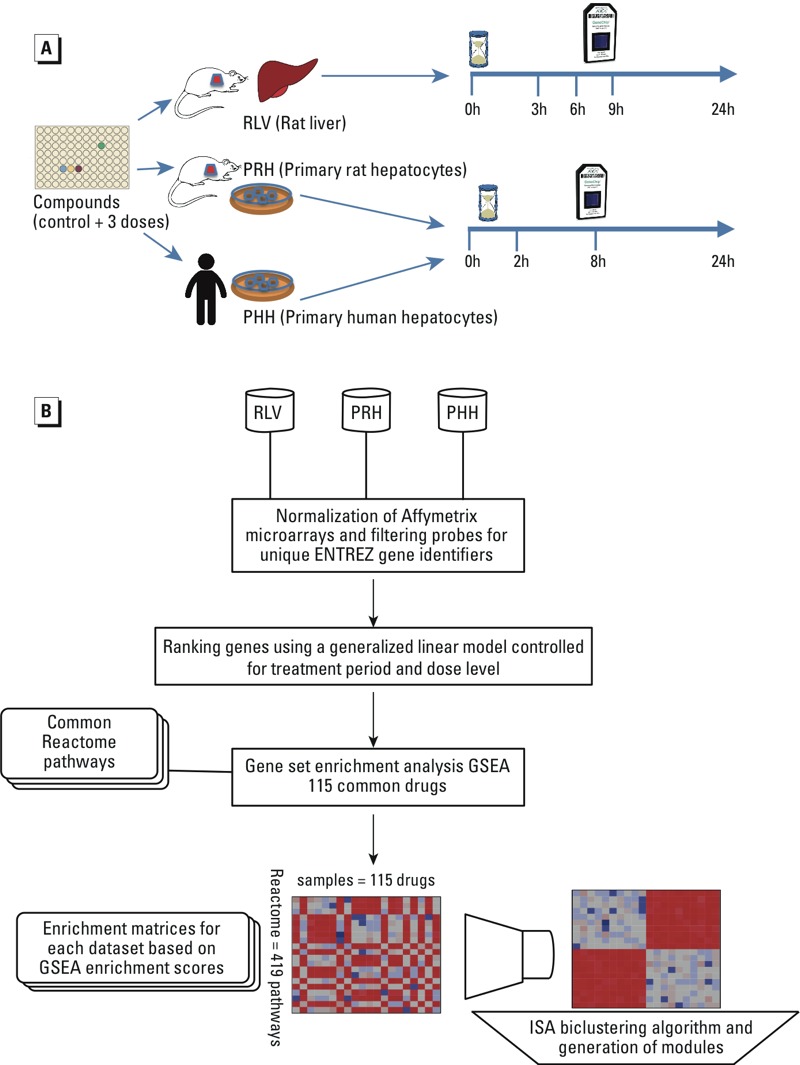
Analysis workflow for the TG-GATEs data set. (*A*) Overview of the TG-GATEs experimental design. TG-GATEs includes rat liver *in vivo* (RLV), rat hepatocyte *in vitro* (PRH), and human hepatocyte *in vitro* (PHH) experiments to test transcriptional responses for > 100 chemical compounds. Samples have been treated with three doses of chemical alongside a control group, and gene expression was measured repeatedly within 24 hr (h) as shown. (*B*) Pathway-based analysis pipeline. A comparative analysis of the three TG-GATEs experiments was conducted by investigating chemical-induced pathways in RLV, PRH, and PHH. For each chemical, a linear regression model was fitted for every gene to assess the effects of the chemical on gene expression, taking into account the treatment period and the dose. Based on these association models, genes were ranked to perform a gene set enrichment analysis (GSEA) on common Reactome pathways. From the enrichment results, transcriptional modules conserved across experimental settings (RLV, PRH, and PHH) were identified by biclustering.

The overall design of our analysis is presented in Figure 1. The three experimental settings that we investigated in TG-GATEs were rat liver *in vivo* and rat and human primary hepatocyte *in vitro* and are referred to as RLV, PRH, and PHH, respectively.

*Microarray retrieval and preparation.* Rat liver and primary rat and human hepatocyte microarray data files were downloaded from ArrayExpress (https://www.ebi.ac.uk/arrayexpress/). The three studies with the accessions E-MTAB-799, E-MTAB-798, and E-MTAB-797 contain toxicogenomic data for RLV, PRH, and PHH experiments, respectively, for > 100 chemical compounds (Figure 1A). PHH and PRH were treated with each compound in duplicate, using three doses (low, middle, and high doses) for three different lengths of time (2, 8, and 24 hr; Figure 1A). Rat liver samples were obtained from animals treated with each compound in triplicate and sacrificed at 3, 6, 9, and 24 hr after dosing (Figure 1A). The highest dose refers to the maximally tolerated dose. Each compound is associated with a corresponding vehicle control for all experimental conditions.

All CEL files (Affymetrix data format that contains the raw intensity values for both perfect match and mismatch probes) were checked for duplicated names and inconsistencies. It was noted that data were missing from human hepatocytes treated with the low dose of 71 chemicals; these 71 chemicals were nevertheless retained and analyzed along with the other 48 chemicals. In total, the transcriptional effects of 119 chemicals on human hepatocytes were obtained from 2,004 microarrays (Affymetrix GeneChip Human Genome U133 Plus 2.0 platform; Affymetrix, Inc.). Similarly, the effects of 129 chemicals on rat liver samples and rat hepatocytes were obtained from 6,192 and 3,096 microarrays, respectively (Affymetrix GeneChip Rat Genome 230_2.0; Affymetrix, Inc.) (Figure 1B). All data sets, including kidney samples in E-MTAB-799 and the repeated dose study (accession E-MTAB-800), were downloaded and curated on the fly through our fully automated pipeline. Documented code is available on GitHub (https://github.com/bhklab/TGGATES).

*Gene expression data.* Gene expression data were normalized with the robust multiarray average (RMA) algorithm (Irizarry et al. 2003) using the Bioconductor BufferedMatrixMethods package (version 1.30.0) (Gautier et al. 2004). Probes were mapped to Entrez Gene IDs using the Bioconductor annotation packages hgu133plus2.db (v3.0.0; Carlson 2015a) and rat2302.db (v3.0.0; Carlson 2015b) for human and rat, respectively. In case of multiple probes mapped to the same Entrez Gene ID, we used the Bioconductor genefu package (v1.15.0) to select the most variant probe set for each gene. This procedure yielded 20,590 and 14,462 unique genes for human and rat, respectively.

*Pathway collections.* Every gene in the curated microarray experiments in TG-GATEs was assigned to pathways described in the Reactome database (Croft et al. 2014) using the Bioconductor BiomaRt package (v2.22.0), for both rat and human genes present in the microarray platform. Pathway collection was performed on 5 March 2014. We subsequently selected the common pathways for rat and human, and we retained only gene sets of sizes between 15 and 500 genes, which resulted in 419 common Reactome pathways for the GSEA analysis (see Supplemental Material, Figure S1). For reproducibility, all curated pathways were stored in gmt files online (https://github.com/bhklab/TGGATES).

*Gene–chemical associations.* Gene ranking was based on gene–chemical associations, which were identified by fitting linear models to estimate the effects of chemical dosage on gene expression controlled by treatment time and interaction between dosage and time. For each pair of gene *i* and chemical *j*, we used the following model:

*G_i_ =* β*_0_* + β*_1_D_j_ +* β*_2_T_j_ +* β*_3_D_j_T_j_*, [1]

where *G_i_* denotes the expression value of gene *i*; *D_j_* is the dose of chemical *j*; *T_j_* is the treatment time for chemical *j*; β*_0_* is the intercept; and β*_1_*, β*_2_*, and β*_3_* are the regression coefficients for the chemical dosage, treatment time, and interaction term of dose and treatment, respectively. The strength of the linear gene–chemical association is given by β*_1_,* and its significance (*p*) is computed using Student’s *t*-test as provided by the lm() function in R (R Core Team 2013).

*Pathway–chemical associations.* Pathways that were significantly perturbed by each chemical were identified using the java implementation of GSEA (v2.0.14) (Subramanian et al. 2005) provided by the Broad Institute. For each chemical, we first ranked all genes with respect to the signed significance of their gene–chemical association: that is, sign(β*_1_*) × –log_10_(*p*), as in Equation 1. We then used each chemical-specific ranked list of genes to perform a preranked GSEA to calculate normalized enrichment scores (NESs) for all common pathways between human and rat. The higher the absolute value of the NES, the more enriched was the corresponding pathway in genes whose expression was significantly perturbed by the chemical of interest. We repeated this process for each chemical and created an “enrichment matrix” with pathway enrichment scores (rows) and chemicals (columns) for each data set (Figure 1B).

*Conserved transcriptional modules.* One hundred and fifteen chemical compounds were common to all three experimental settings (Figure 1B; see also Supplemental Material, “Common list of chemicals: One hundred and fifteen common chemicals analyzed in the TG-GATEs project”). For each of these data sets, we applied a biclustering method: that is, we applied the iterative signature algorithm (ISA) (Bergmann et al. 2003) implemented in the isa2 package (v0.3.3) (Csárdi et al. 2010) in R (R Core Team 2013) to the enrichment matrix to simultaneously identify similar biochemical-induced transcriptional response patterns. The ISA algorithm runs with all combinations of threshold values on rows and columns, which has been described in detail on the TG-GATEs companion website (https://www.pmgenomics.ca/bhklab/pubs/tggates). Similarly to Iskar et al. (2013), we merged modules with similar sets of pathways using the isa.unique() function in the isa2 package to filter redundant modules using a correlation limit of 0.5 to determine redundant biclusters. Lastly, modules sharing common sets of pathways and common chemicals across the different data sets—namely RLV, PRH, and PHH (inter–data set similarity)—were identified using a one-sided hypergeometric test (*p* < 0.001); this technique is referred to as the reciprocal best-hit approach (Iskar et al. 2013).

*Reproducible research.* To ensure full reproducibility, this work complied with the guidelines proposed by Robert Gentleman (Gentleman 2005) in terms of the availability of the code and the reproducibility of results and figures. The procedure to properly set up the software environment and run our analysis pipeline is provided in Supplemental Material, “Reproducibility of analysis.” The analysis code is also publicly available at https://github.com/bhklab/TGGATES.

## Results

The approach we used to investigate the pathways altered by chemical perturbations leverages the transcriptional profiling data available in TG-GATEs for RLV, PRH, and PHH, as summarized in Figure 1A. We analyzed each of these three data sets separately and compared the results from the *in vitro*–treated hepatocytes (PRH and PHH) with those from the livers of treated rats (RLV) because this animal model is considered to be the gold standard in toxicity studies. Preprocessing of these gene expression data sets yielded sets of 20,590 and 14,460 unique genes from the human and rat microarray platforms, respectively, that were kept for subsequent analysis. The association between gene expression and the 115 chemicals that were common to the three TG-GATEs experimental settings (PRH, PHH, and RLV) was then investigated at the pathway level using the preranked version of GSEA (Subramanian et al. 2005). A total of 419 pathways that were common to both rat and human were queried from the Reactome database to identify pathways that were modulated upon chemical perturbation (see Supplemental Material, Figure S1). Matrices containing the enrichment scores of each pathway perturbed by each chemical were then analyzed using an unsupervised biclustering technique, ISA (Bergmann et al. 2003), to define functional modules (i.e., clusters of pathways) specifically associated with diverse chemical treatments. Each module was given a summary name, that is, a Reactome parent term that best recapitulated the pathways enriched in the module (see Supplemental Material, Table S1).

*Conservation of transcriptional modules across experimental settings.* Rat liver *in vivo* treated with a single dose. Twenty-four nonredundant modules were identified using the aforementioned ISA analysis (*p* < 0.001). These modules were enriched for the following biological pathways: neuronal system; hemostasis; cell cycle checkpoints; DNA repair; mitosis; lysosome disorders; innate immune system; NOTCH, transforming growth factor beta receptor (TGF-βR)/SMAD, and phosphoinositide 3-kinase/protein kinase B (PI3K/AKT)signaling cascades; lipid metabolism; and mitochondrion-dependent processes. The summary names of all modules are provided in Supplemental Material, Table S1.

**Figure 2:**
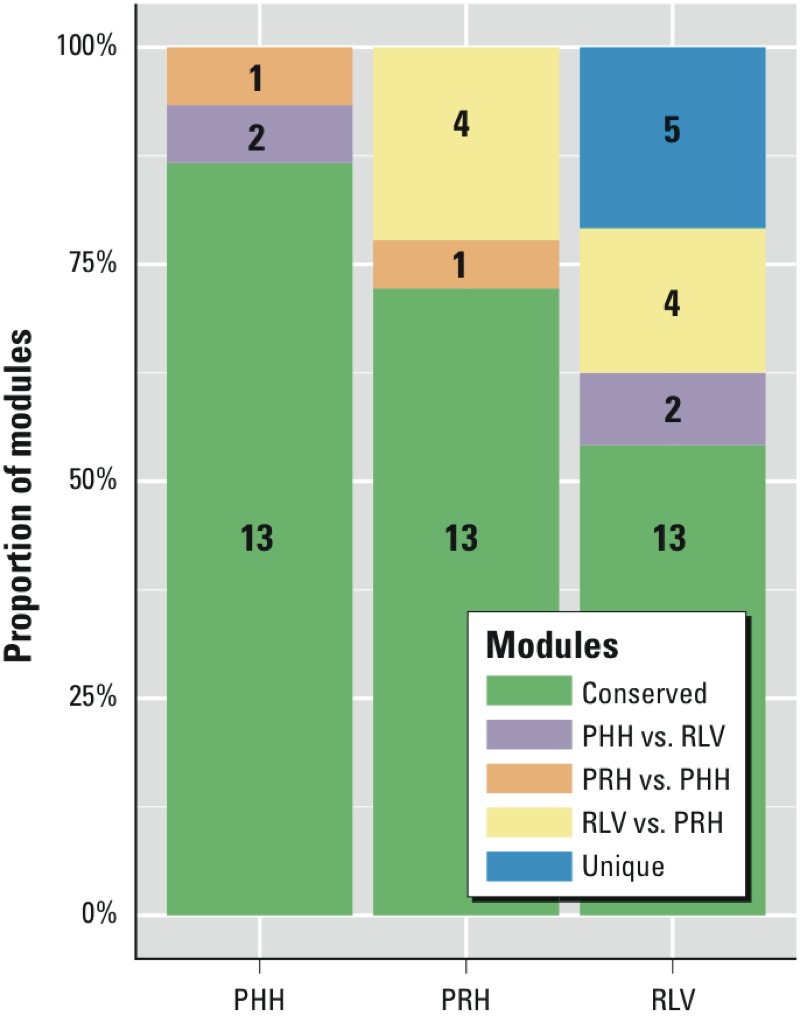
The number of non-redundant transcriptional modules and proportions identified for each and across all experimental settings in TG-GATEs. Each bar corresponds to an experimental setting in TG-GATEs (RLV, PRH, PHH) and contains the number of modules found to be unique for the experiment (blue) or shows a corresponding module in another experiment (see color legend: green for conserved, purple for conserved in RLV vs. PHH, orange for conserved in PHH vs. PRH, yellow for conserved in RLV vs. PRH). Although only a few modules were detected in only one or two settings, most modules showed significantly high overlap in terms of pathway enrichment across all settings (hypergeometric *p *< 0.001).

Primary rat hepatocytes versus rat liver *in vivo*. The ISA algorithm detected 18 modules in PRH. Interestingly, 17 modules overlapped with RLV using a reciprocal best-hit approach in which 2 modules were considered to be conserved if their Reactome pathways significantly overlapped (Iskar et al. 2013) (hypergeometric *p* < 0.001). Only 1 module related to cholesterol biosynthesis did not overlap at the specified cutoff. Figure 2 shows in detail the number of nonredundant ISA modules in each data set and their conservation across the experimental settings.

Primary human hepatocytes versus rat liver *in vivo*. ISA analysis resulted in the identification of 15 modules in PHH toxicogenomic data. Again, all but one (i.e., 14 modules) overlapped with RLV (hypergeometric *p* < 0.001; Figure 2).

**Figure 3:**
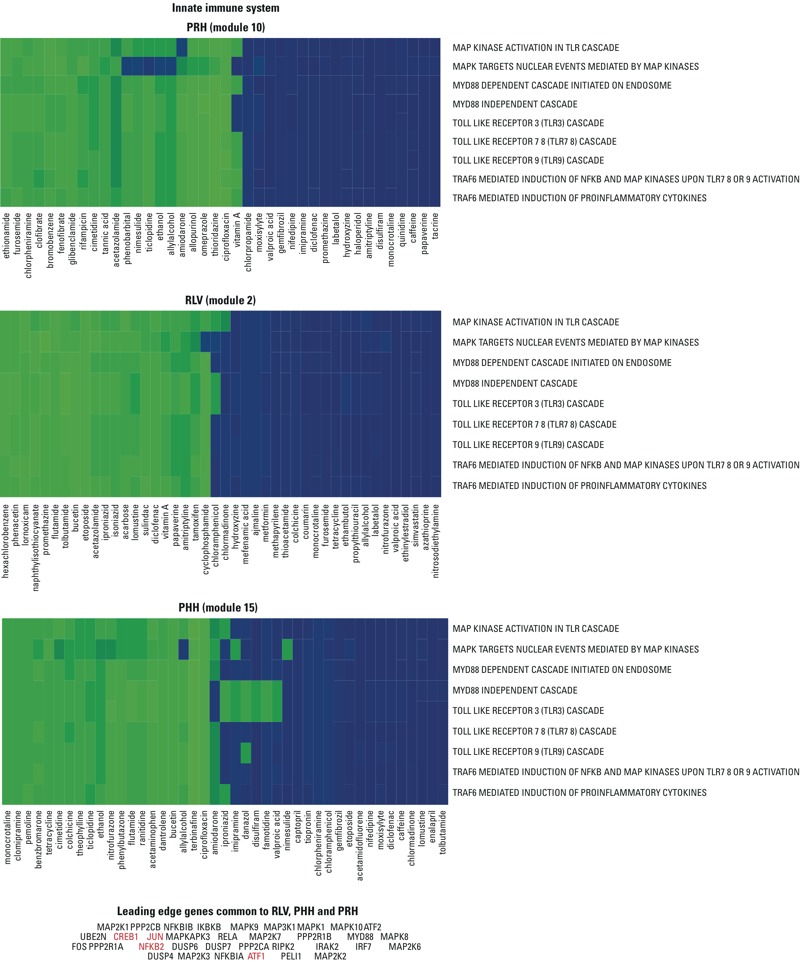
Conservation of modules across *in vitro* and *in vivo* settings based on Reactome pathways. This example summarizes a conserved module between RLV, PRH, and PHH, shown as heatmaps and keeping overlapping pathways colored with respect to their enrichment scores: up-regulated pathways are shown in blue, and down-regulated pathways are shown in green. The three heatmaps correspond to a conserved module associated with the innate immune system (mod2 in RLV, mod15 in PHH, and mod10 in PRH). The leading edge genes from common pathways that are****activated or repressed by chemicals are shown under the heatmap with known oncogenes colored in red. (For more details, see Supplemental Material, Zip files S2 and S4.)

Overall, we identified 13 modules that were conserved across the three experimental setting data sets (RLV, PHH, and PRH) (see Supplemental Material, Table S1 and Figure S2). As a representative example, we show a conserved module in Figure 3. The module is enriched for components of the innate immune system, and the overlapping pathways are in the same order for RLV, PHH, and PRH. We extracted the union of the genes that were found to contribute to the enrichment score [referred to as “leading edge” (Subramanian et al. 2005)] of at least one pathway for all chemicals in the module. From this union, we obtained a list of common genes that were activated or repressed by chemical stress in RLV, PRH, and PHH. Heatmaps for all ISA settings, lists of hypergeometric *p*-values, and lists of leading edge genes are provided in separate Supplemental Material, Zip files S2, S3, and S4.

**Figure 4:**
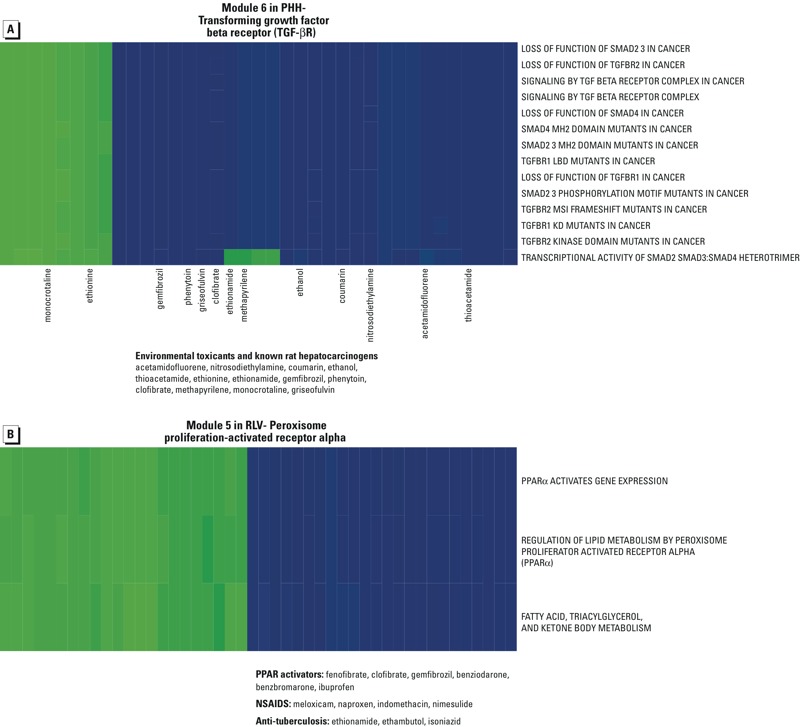
Characterization of putative biomarkers within chemical-induced modules. (*A*) Heatmap representing a module in PHH (mod6), associated with transforming growth factor beta receptor signalling, that can be considered as a candidate biomarker in humans for environmental exposure to known toxicants. Diverse rat hepatocarcinogens were enriched in this module. (*B*) Heatmap representing a module in RLV (mod5) that was relevant to toxicity mode of action and is enriched for a class of lipid-lowering drugs known as fibrates. These drugs are rat hepatocarcinogens and activate the peroxisome proliferation-activated receptor alpha (PPARα). Green, down-regulation; blue, up-regulation. Drugs that activate PPAR pathways include nonsteroidal anti-inflammatory and antituberculosis drugs. All statistical details and genes contributing to these pathways are in Supplemental Material, Zip file S4.

*Enrichment for hepatocarcinogens*. The approach described above identified 13 modules associated with the early response of hepatocytes to diverse chemicals that are conserved *in vivo*, *in vitro,* and between rat and human. To test whether some modules were significantly associated with the hepatocyte response to known hepatocarcinogens, we investigated 25 previously validated rat hepatocarcinogens (Yamada et al. 2013) present among the 115 chemicals investigated in our study (see Supplemental Material, “Common list of chemicals: One hundred and fifteen common chemicals analyzed in the TG-GATEs project”). Specifically, these hepatocarcinogens were significantly enriched in the NOTCH and TGF-βR/SMAD signaling modules in PHH (hypergeometric *p* < 0.05), but not in PRH or RLV. The TGF-βR/SMAD signaling module (Figure 4A) in PHH was enriched for known environmental toxicants and carcinogens (e.g., ethionine, thioacetamide, coumarin, ethanol, 2-acetamidofluorene, *N-*nitrosodiethylamine). None of these modules was enriched for hepatocarcinogens in RLV, and this was only the case for the PI3K/AKT module in PRH (*p* = 0.049; see Supplemental Material, Zip file S3). The known rat hepatocarcinogens were also significantly associated with the neuronal system/G protein-coupled receptors (GPCRs) module in both RLV and PHH, but not in PRH, likely reflecting the pleiotropic roles that GPCRs play in many cellular processes, including chemical carcinogenesis (see Supplemental Material, Zip files S2 and S3).

As a control experiment, we selected 12 noncarcinogenic compounds (see Supplemental Material, “Common list of chemicals: One hundred and fifteen common chemicals analyzed in the TG-GATEs project”) and determined whether they were significantly associated with any of the modules in RLV, PRH, and PHH. As anticipated, no enrichment was observed, particularly for those modules enriched for known hepatocarcinogens in PHH. As an additional control, we ascertained that the NOTCH and TGF-βR/SMAD modules were indeed enriched in cancer-related pathways by showing that the 20 pathways containing the word “cancer” in the Reactome common data set (out of 419 pathways in total) were in fact enriched in those modules (hypergeometric *p* < 0.001). This enrichment did not occur for any of the remaining modules without cancer terms. Collectively, the results presented above support that primary human hepatocytes can detect potential environmental chemical carcinogens (Figure 4A). By extension, we inferred that the other modules were also enriched in pathways pertinent to chemical exposure.

*Activation of the peroxisome proliferator activated–receptor alpha*. Because some peroxisome proliferator activated–receptor alpha (PPARα) activators are known to induce hepatocarcinogenesis in rodent liver, we investigated whether PPARα activators (e.g., benziodarone, benzbromarone, fenofibrate, clofibrate, ibuprofen, WY-14643, gemfibrozil) were randomly distributed across modules in RLV, PHH, and PRH. Interestingly, none of the modules in PHH or PRH was enriched for those drugs; however, we found that a module unique to RLV was significantly associated with the regulation of lipid metabolism by PPARα and was enriched for those drugs (*p* = 0.014). Other potential PPARα inducers were found in this module, including nonsteroidal anti-inflammatory (NSAIDs) and antituberculosis drugs (Figure 4B).

A recent study (Grinberg et al. 2014) showed that numerous compounds from TG-GATEs cause “stereotypical” transcriptional responses in PHH. This term is used to describe a response wherein a cytotoxic concentration of numerous compounds causes a consensus expression response regardless of the chemical class of compound. For each module, we assessed the significance of the overlap between all leading edge genes, which we generated from the biclustering in PHH, and the genes deregulated by at least 20 compounds in the study by Grinberg et al. (2014). We demonstrated that stereotypical clusters of genes involved in liver metabolic functions and cell proliferation were enriched in two modules from PHH, mainly those associated with normal liver function and DNA synthesis. Furthermore, to ascertain that our observations from PHH were not simply experimental artifacts due to *in vitro* conditions, we selected liver cirrhosis as a case study and tested the enrichment for genes associated exclusively with liver cirrhosis in PHH (Grinberg et al. 2014). Interestingly, the TGF-βR signaling module in PHH (module 6) was significantly enriched for genes linked to liver cirrhosis in addition to being induced by known hepatocarcinogens and environmental toxicants (Figure 4B).

Finally, we demonstrated the similarity of the distributions of genes perturbed by rat hepatocarcinogens and nonhepatocarcinogens (see Supplemental Material, Figure S2).

## Discussion

We tested the extent to which transcriptional responses associated with liver toxicity can be recapitulated across human and rat and between *in vivo* and *in vitro* settings. To do so, we exploited toxicogenomic information generated by the TG-GATEs project from liver samples of rats treated with different chemicals and from rat/human hepatocytes exposed to the same compounds *in vitro*. To date, several studies have used TG-GATEs to build predictors of relevant toxicological endpoints. For example, Zhang et al. recently used these data to build a predictive gene signature for both hepatotoxicity and nephrotoxicity (Zhang et al. 2014). Interestingly, Zhang et al. (2014) revealed the importance of early response genes in triggering toxicity-associated signaling networks, as highlighted by the high predictive power of the signature derived from a treatment period of less than 24 hr.

To our knowledge, our study is the first analysis of the TG-GATEs data comparing functional changes—in the form of transcriptional responses—induced by a large panel of chemicals *in vivo* (rat liver), *in vitro* (cultured hepatocytes), and across species (human vs. rat). A major feature of our approach is the fact that it relied on a pathway enrichment analysis, thereby allowing comparisons to be made between species without the need to rely on a limited subset of orthologous genes. In this context, it is worth contrasting our findings with those of Iskar et al. (2013), who identified, solely on the basis of orthologous genes, transcriptional modules that were conserved between rat liver (Natsoulis et al. 2008) and three human cancer cell lines from the Connectivity Map (CMap) (Lamb et al. 2006). The findings of Iskar et al. (2013) revealed that 15% of the chemical-induced modules were conserved across cell lines and species. However, this approach was limited to 8,962 genes in CMap, which corresponded to only 3,618 orthologous genes available for the rat liver experiments. To overcome this limitation, by focusing on common pathways between species, our approach enabled a full exploration of the TG-GATEs data sets and allowed the identification of functional pathways altered by chemical treatments in both rat and human.

Our results indicated that the response of hepatocytes to chemical insults is analogous *in vitro*, *in vivo*, and across human and rat in that it involves a conserved set of cellular pathways. Specifically, we identified 13 highly conserved modules representative of the early response of hepatocytes to chemical exposure. Two of those modules are enriched in key signalling pathways associated with cancer, namely the TGF-βR superfamily module (TGF-βR–mod17 in RLV) and the NOTCH signaling module (NOTCH–mod6 in RLV). Given the roles that the TGF-βR and NOTCH pathways play in response to early toxicity (Zhang et al. 2014) and in maintaining normal liver function (Morell and Strazzabosco 2014), respectively, it was not surprising that these modules were enriched for known rat hepatocarcinogens, including environmental toxicants. What is more puzzling, given our results, is the fact that these two pathways are significantly associated with hepatocarcinogens only in humans and not in rats. This disparity may reflect a key difference in how both species handle these chemicals. That the responses of rats and humans may differ for some chemicals is further supported by our finding that the PPARα agonists clofibrate, fenofibrate, gemfibrozil, benziodarone, and benzbromarone up-regulate pathways associated with PPARα activation only in rat liver, thus providing a potential mechanism underlying the hepatocarcinogenicity of these drugs in rats but not in humans (Lai 2004).

Several lines of evidence suggest that the modules identified in the present study are relevant to how hepatocytes respond to chemicals. For example, one of the modules we identified, the innate immune system (mod2 in RLV), was enriched in proinflammatory Toll-like receptor signaling pathways, which were shown by Huang et al. to be good predictors of drug-induced liver injury (Huang et al. 2010). Our results are also consistent with those reported in comparative studies by Doktorova et al., who assessed the transcriptional profiles of toxicants for rat liver and a panel of *in vitro* models (Doktorova et al. 2012, 2013). Those studies assigned deregulated genes by performing *in vivo*/*in vitro* comparison. Moreover, we found that pathways associated with G protein-coupled receptors (GPCRs) and the neuronal system were consistently affected by a variety of chemicals. Of particular relevance is the fact that some chemicals found in this conserved module (neuronal system–mod8 in RLV) can cause the potentially lethal long QT syndrome (delayed repolarization of the heart) by perturbing heart conductance. For example, ciprofloxacin, haloperidol, thioridazine, quinidine, and amiodarone are well known to prolong the QT interval and to cause *torsades de pointes,* a deadly form of arrhythmia (Fazio et al. 2013). This module was also enriched for known rat hepatocarcinogens in RLV and PHH but not in PRH, a finding that may relate to the fact that ion channels, in addition to being involved in long QT syndrome, can also play a role in carcinogenesis (Babcock and Li 2013). However, this observation might not be specific to a particular class of compounds because the Reactome pathways related to the neuronal system contain a large number of genes (> 500). Our findings also suggest that some chemicals modulate pathways associated with vitamin metabolism (metabolism of vitamins and cofactors–mod3 in RLV) in hepatocytes, in particular those associated with the inherited metabolic disorders methylmalonic aciduria and homocystinuria. Surprisingly, the scientific literature contains only a few reports pertaining to the association between chemical-induced liver injury and vitamins. Among the studies that we found relevant to this work, one describes an association between high levels of circulating cobalamin (vitamin B_12_) and several serious liver diseases (Ermens et al. 2003), and another highlights the role of vitamin B_12_ metabolism in methylmalonic aciduria, a disorder that can lead to severe liver injury and necessitate, in some cases, liver transplantation (Hansen and Horslen 2008). Given the strong association between vitamin metabolism and early drug exposure that has been revealed in our study, it may be of interest to explore this understudied area of research in greater depth.

Furthermore, we confirmed the biological relevance of our biclusters against findings from a recent study (Grinberg et al. 2014). Indeed, we showed that our modules recapitulated stereotypical response to chemicals as well as compound-specific perturbations. Moreover, we found evidence that the TGF-βR signaling module in PHH could act as a potential biomarker of chemical injury that may lead to liver cirrhosis in addition to being enriched for known hepatocarcinogens.

It is worth noting that our new bioinformatics pipeline complements previous approaches that have been used to elucidate mechanisms of chemical toxicity *in vitro* or *in vivo* by enabling efficient and unbiased exploration of chemical-induced transcriptional changes in both types of systems and across species. The modules that emerged from this analysis suggest that functional networks of xenobiotic detoxification and response to external stress are highly conserved in the hepatic system across human and rat. In contrast to pathway conservation, our results suggest that the chemicals associated with any given module do not show a meaningful overlap between *in vitro* and *in vivo* systems or across species. Although this concept seems somewhat counterintuitive, it has been observed previously (Zhang et al. 2014) and may reflect genuine differences between systems in chemical bioactivation through metabolism, thus complicating the interpretation of *in vivo* versus *in vitro* data. Another factor that must be considered when assessing the value of our approach is the fact that it relied on an expert knowledge–curated, peer-reviewed database of functional pathways. Although the database provided an alternative resolution for the orthologous gene limitation, we are nevertheless aware that annotations in pathway databases are incomplete and thus may limit this approach to some extent. Some of these limitations may be addressed in the future as we extend our approach to other systems (e.g., the HepG2 hepatocellular carcinoma cell line) and to other toxicogenomic databases, such as DrugMatrix (Natsoulis et al. 2008), and as we integrate more “omics” data, including data from RNA sequencing and single nucleotide polymorphism profiling, to take into account the variability of individual responses to chemicals.

## Conclusion

Our analysis of the TG-GATEs data presented herein indicates that toxicogenomics-based cellular models recapitulate most of the pathways related to chemical-induced injury in rat liver. Furthermore, it may be possible to reduce unnecessary animal testing in early toxicological assessments by complementing animal experiments with *in vitro* testing. Because environmental toxicants can be associated with alterations in cellular pathways that contribute to general injury patterns and likely to more severe phenotypes such as carcinogenesis, the TGF-βR/SMAD module could serve as a putative biomarker to identify chemicals with carcinogenic potential for humans. Notably, potent carcinogenic compounds such as 2-acetamidofluorene, *N-*nitrosodiethylamine, and ethanol were found in this module in PHH.

Our findings could be generalized to study a large set of environmental contaminants relevant to human health. Therefore, our method can help to identify numerous pathways and genes associated with chemical-induced toxicity.

## Supplemental Material

(116 KB) PDF
