# Supplementary material for: Characterization of Conserved Toxicogenomic Responses in Chemically Exposed Hepatocytes across Species and Platforms
Source: Environ Health Perspect. 2015 Jul 14;124(3):313–20. doi: 10.1289/ehp.1409157 (PMC4786983; doi:10.1289/ehp.1409157)
Supplement: (116 KB) PDF [file ehp.1409157.s001.acco.pdf]

Environ Health Perspect
DOI: 10.1289/ehp.1409157

Note to Readers: *EHP* strives to ensure that all journal content is accessible to all readers. However, some figures and Supplemental Material published in *EHP* articles may not conform to 508 standards due to the complexity of the information being presented. If you need assistance accessing journal content, please contact ehp508@niehs.nih.gov. Our staff will work with you to assess and meet your accessibility needs within 3 working days.

Supplemental Material

Characterization of Conserved Toxicogenomic Responses in Chemically Exposed Hepatocytes across Species and Platforms

Nehme El-Hachem, Patrick Grossmann, Alexis Blanchet-Cohen, Alain R. Bateman, Nicolas Bouchard, Jacques Archambault, Hugo J.W.L. Aerts, and Benjamin Haibe-Kains

Table of Contents

All supplemental material are being hosted and maintained at a companion website https://www.pmgenomics.ca/bhklab/pubs/tggates/. This Table of Contents contains all the supplemental materials referenced within our manuscript. These are listed below:

Supplemental Material – Table 1: This table shows an approximated parent term extracted from the Reactome database for the conserved modules in RLV, PHH and PRH. Modules in all datasets are highlighted according to the following color scheme: Blue: module unique to one experimental setting (RLV, PRH or PHH); Yellow: conserved between RLV and PRH; Orange: conserved between PRH and PHH; Violet: conserved between PHH ad RLV; and Green: conserved in all three experimental settings.

https://www.pmgenomics.ca/bhklab/sites/default/files/downloads/table1.pdf

Supplemental Material – Figure S1: Venn diagram showing the overlap between human and rat Reactome pathways.

https://www.pmgenomics.ca/bhklab/sites/default/files/downloads/Figure_S1.pdf

Supplemental Material – Figure S2: Zip file with all the histograms showing the distribution of significant differentially expressed genes in hepatocarcinogens vs. non hepatocarcinogens, in RLV, PRH and PHH respectively (False discovery rate < 10%).

https://www.pmgenomics.ca/bhklab/sites/default/files/downloads/Figure_S2.zip

Supplemental Material – Common list of chemicals: **One hundred and fifteen common chemicals analyzed in the TG-GATEs project.** Among the experiments in TG-GATEs, these 115 chemicals were common for the rat *in vivo*, primary human hepatocytes, and primary rat hepatocytes platforms. This included known rat hepatocarcinogens. Non-carcinogenic compounds, selected as a negative control, are highlighted in blue.

https://www.pmgenomics.ca/bhklab/sites/default/files/downloads/chemicals.pdf

Supplemental Material – S2: Zip file of all module heatmaps in RLV, PHH and PRH.

https://www.pmgenomics.ca/bhklab/sites/default/files/downloads/S2.zip

Supplemental Material – S3: Zip file with xls files containing p-values of module overlaps (for all experimental settings), p-values for the 'special' cases such as hepatocarcinogens/cancer pathways/etc.

https://www.pmgenomics.ca/bhklab/sites/default/files/downloads/S3.zip

Supplemental Material – S4: Zip file with all leading edge genes in all modules for all datasets.

https://www.pmgenomics.ca/bhklab/sites/default/files/downloads/S4.zip

Supplemental Material – Reproducibility of analysis: Document describing how to reproduce the study results by running the analysis pipeline.

https://www.pmgenomics.ca/bhklab/sites/default/files/downloads/analysis.pdf

All codes and R scripts are found on: https://github.com/bhklab/TGGATES

Normalized microarray data can be automatically obtained by running the pipeline, and these data are also available from here:

https://www.pmgenomics.ca/bhklab/sites/default/files/downloads/TGGATES_normalized_data.zip
